# Association between NEAT1 polymorphism and the risk of lung cancer: A protocol for systematic review and meta-analysis

**DOI:** 10.1097/MD.0000000000025478

**Published:** 2021-04-23

**Authors:** Zhongbao Hu, Jingsheng Chen, Ping Meng, Ming Li

**Affiliations:** Department of Oncology, E’zhou Central Hospital, E’zhou, Hubei Province, China.

**Keywords:** long non-coding RNA, lung cancer, meta-analysis, nuclear paraspeckle assembly transcript 1, polymorphism, protocol

## Abstract

**Background:**

Long noncoding RNAs play vital roles in development and progression of lung cancers. Nuclear paraspeckle assembly transcript 1 (NEAT1) polymorphisms were reported to be closely related to lung cancer susceptibility. Recently, numerous studies have been performed to detect the association between NEAT1 polymorphisms and lung cancer susceptibility. However, their results were inconsistent and controversial. So, we carried out a meta-analysis aiming to define the association exactly.

**Methods:**

Appropriate studies were retrieved from searching Web of Science, PubMed, Scopus, and Google scholar databases, updated January 31, 2021. The pooled odds ratios with 95% confidence intervals were calculated to estimate the strength of the association between NEAT1 polymorphisms and lung cancer risk. All of the data were analyzed with Stata 16.0.

**Results:**

The results of this meta-analysis will be submitted to a peer-reviewed journal for publication.

**Conclusion:**

This meta-analysis will summarize the relationship between NEAT1 polymorphism and lung cancer.

## 1 Introduction

Lung cancer is the highest incidence of malignant tumor in the world.^[1,2]^ The etiology of lung cancer is still not completely clear. Smoking is a confirmed environmental risk factor of lung cancer. Although smoking is closely associated with lung cancer, it is estimated that half of the new cases come from people who never smoked or quitted for many years. Other studies have displayed that about one-fourth of lung cancer cases are nonsmokers.^[3]^ In addition, epidemiological studies have revealed that, in addition to environmental factors, susceptibility to lung cancer is a key genetic factor.^[4–6]^

Noncoding RNA is a kind of RNA molecule whose length is more than 200nt and lacks the ability of protein coding.^[7]^ Studies have found that long noncoding RNA (lncRNA) can affect the growth and apoptosis of many kinds of cancer cells as well as the progression and metastasis of cancer.^[8,9]^ A large number of studies have also proved that lncRNA is mal-expressed in a variety of tumors, including gastric cancer, liver cancer, colorectal cancer, bladder cancer, and so on.^[10–13]^

As the most common genetic variation, single-nucleotide polymorphism has attracted more and more attentions in cancer research. According to previous studies, it has been found that single nucleotide polymorphism can predict the risk and prognosis of cancer.^[14,15]^ Long non-coding RNA nuclear paraspeckle assembly transcript 1 (NEAT1) is a nuclear-enriched lncRNA, and about 3.7 kb located on chromosome 11q13.1.^[16]^ Previous studies have illustrated that NEAT1 has tumor marker significance in colorectal cancer, breast cancer, and other tumors.^[17,18]^ Studies have demonstrated that single-nucleotide polymorphisms in NEAT1 are related to the occurrence of cancer.^[19,20]^

At present, there are many studies on the relationship between NEAT1 polymorphism and lung cancer susceptibility, whereas the conclusions are very different. In this article, meta-analysis was conducted to systematically and quantitatively analyze the results of published case-control studies on the relationship between single nucleotide polymorphism of NEAT1 and the risk of lung cancer, so as to provide evidence-based medical evidence for lung cancer susceptibility.

## 2 Methods

### 2.1 Study registration

The protocol of this review was registered in OSF (OSF registration number: DOI 10.17605/OSF.IO/JRSY6). It was reported to follow the statement guidelines of preferred reporting items for systematic reviews and meta-analyses protocol.^[21]^

### 2.2 Inclusion criteria

All studies were included based on the following criteria: case–control study and the evaluation of the association between NEAT1 polymorphisms and lung cancer susceptibility.

### 2.3 Exclusion criteria

The exclusion criteria are as follows: duplication publication with the same population and no available data even contracted with authors.

### 2.4 Publication search

Two investigators independently performed a systematically computerized search for English studies through PubMed, Google scholar, EmBase, and Web of Science databases up to January 31, 2021. The keywords applied are as follows: “long noncoding RNA nuclear paraspeckle assembly transcript 1, lncRNA NEAT1 or long non-coding NEAT1,” “polymorphisms, variants, or variation,” and “lung cancer, lung carcinoma, lung tumor, or lung neoplasm.” Furthermore, studies were identified through manual searches on reviews and retrieved studies. The search strategy for PubMed is illustrated in Table 1, and the corresponding keywords would be applied in other databases.

**Table 1:** Search strategy for PubMed.

Number	Search terms
#1	Lung Neoplasms[MeSH]
#2	Cancer of Lung[Title/Abstract]
#3	Lung Cancer[Title/Abstract]
#4	Pulmonary Cancer[Title/Abstract]
#5	Pulmonary Neoplasms[Title/Abstract]
#6	Cancer of the Lung[Title/Abstract]
#7	Neoplasms, Lung[Title/Abstract]
#8	Neoplasms, Pulmonary[Title/Abstract]
#9	Cancer, Lung[Title/Abstract]
#10	Cancer, Pulmonary[Title/Abstract]
#11	Cancers, Lung[Title/Abstract]
#12	Cancers, Pulmonary[Title/Abstract]
#13	Lung Cancers[Title/Abstract]
#14	Lung Neoplasm[Title/Abstract]
#15	Neoplasm, Lung[Title/Abstract]
#16	Neoplasm, Pulmonary[Title/Abstract]
#17	Pulmonary Cancers[Title/Abstract]
#18	Pulmonary Neoplasm[Title/Abstract]
#19	Carcinoma, Non-Small-Cell Lung[MeSH]
#20	Carcinoma, Small Cell[MeSH]
#21	or/1-20
#22	nuclear paraspeckle assembly transcript 1[Title/Abstract]
#23	NEAT1[Title/Abstract]
#24	or/22-23
#25	polymorph∗[Title/Abstract]
#26	susceptibility[Title/Abstract]
#27	or/25-36
#28	#21 and #24 and #27

### 2.5 Data collection and analysis

#### 2.5.1 Selection of studies

**Figure 1:**
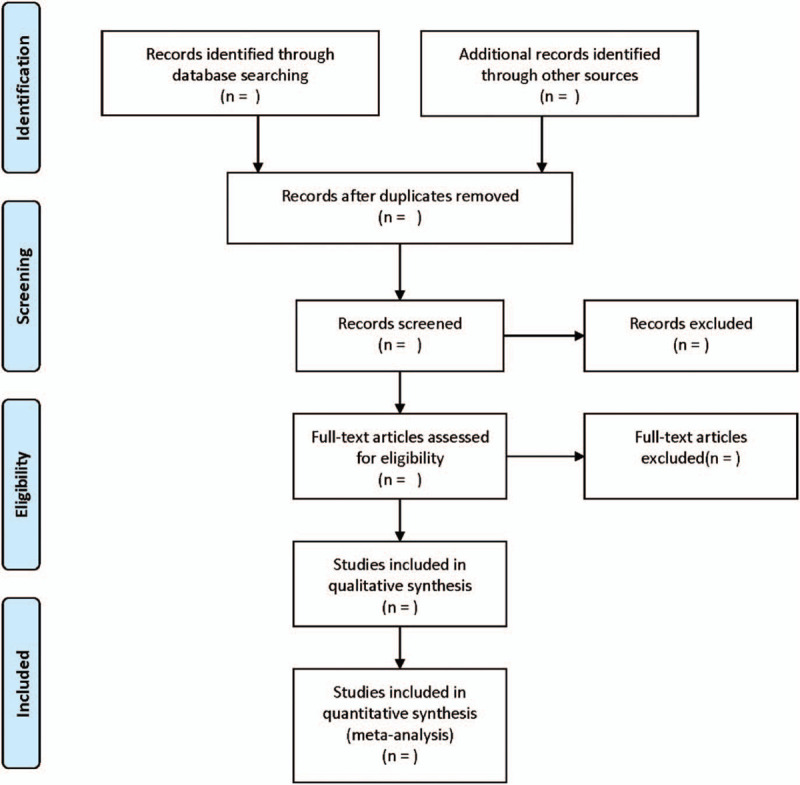
Flow diagram of study selection process.

The 2 reviewers complete the screening process independently, and any differences are decided by a third reviewer. The screening process of the article includes reading the title, the abstract and the full text, so as to determine whether it meets the inclusion criteria. The researchers record the reasons to exclude each study in light of the preferred reporting items for systematic reviews and meta-analysis guidelines and report the screening results as well. The flowchart is exhibited in Figure 1.

#### 2.5.2 Data extraction

Data were extracted by 2 independent investigators from included studies.

Divergence was solved by discussing every item. The collected information from each article was as follows: first author, publication year, region, cases and controls number, source of control, genotype frequencies, genotyping method, and *P* value for Hardy–Weinberg equilibrium (HWE) of controls. Meanwhile, we categorized ethnicity as White, African, or Asian.

#### 2.5.3 Methodology quality assessment

Two investigators independently evaluated the quality of the included articles on the basis of the Newcastle-Ottawa scale (NOS) that is adopted to evaluate the quality of observational studies.^[22]^ The quality score items included the representativeness of the cases, the source of the controls, genotyping examination, HWE in controls and association assessment. The NOS values arrange from 0 to 9. Studies with the score of 6 are considered to be of high quality.^[23]^

#### 2.5.4 Dealing with missing data

The reason for the loss of data in the period of data screening and extraction is identified here. We would attempt to contact the authors if the data of potential studies are insufficient, missing, or vague. These studies would be excluded only if the data are not available through the method described above.

#### 2.5.5 Statistical analysis

The HWE for control subjects of each study was evaluated by carrying out *χ*^2^ test, and *P* < .05 was seen as significant disequilibrium. Odds ratios (ORs) and the 95% confidence intervals (95% CIs) were calculated to evaluate the association of the NEAT1 polymorphism and lung cancer susceptibility. The pooled ORs were executed for homozygote comparison, dominant and recessive models, allele comparison and heterozygote comparison. The heterogeneity was calculated by performing *χ*^2^-based *I*^2^ test and *Q* test. The fixed-effect model (the Mantel-Haenszel method) was chosen when *I*^2^ value is <50%. When *I*^2^ is >50%, a random-effects model (DerSimonian and Laird method) was adopted. All of the statistical analyses were conducted by STATA 16.0 (StataCorp, College Station, TX), and the *P* values were 2-sided.

#### 2.5.6 Subgroup analysis

According to different ethnicity, genotyping method, and so on, we carried out subgroup analyses of the relationship between NEAT1 polymorphisms and the risk of lung cancer.

#### 2.5.7 Sensitivity analysis

The eligible study was sequentially removed to perform the sensitivity analysis.

#### 2.5.8 Assessment of publication biases

Publication bias was assessed by Begg rank correlation and Egger linear regression. The publication bias was regarded to be statistically difference when *P* < .05.^[24,25]^

#### 2.5.9 Ethics and dissemination

The content of this article does not involve moral approval or ethical review and would be presented in print or at relevant conferences.

## 3 Discussion

LncRNA is a kind of functional RNA molecules that are longer than 200nt, and most of them lack the function of coding protein.^[26,27]^ Recent studies have shown that lncRNA plays an important role in tumor proliferation, metastasis, and angiogenesis, and is often involved in tumor regulation as a tumor suppressor gene.^[28,29]^ At the same time, lncRNA has the feature of specific expression in tissue cells, and can be adopted to classify tumor subtypes or to predict the therapeutic effects of drugs.

Previous studies have confirmed that NEAT1 is abnormally expressed in many human malignant tumors, including leukemia, glioma, non-small cell lung cancer, ovarian cancer and breast cancer, and participates in the occurrence, metastasis, and prognosis of tumors.^[30–34]^ The study also revealed that the single nucleotide polymorphism of *NEAT1* gene is related to its expression. For example, rs2239895 polymorphism has enhancer activity, and can also upregulate the expression of NEAT1.^[19]^ In this article, meta-analysis was used to analyze the relationship between multiple mutation sites of NEAT1 and lung cancer susceptibility, which will provide a new idea for the diagnosis and treatment of lung cancer.

This study has the following shortcomings. First, meta-analysis itself is an observational study, so the lack of original experimental data limits further researches, such as the inability to carry out subgroup analysis of different genders and different types of lung cancer. It is impossible to analyze the interaction between two sites. Second, there may exist publication bias, because the literature with positive results is easier to publish. Third, some included loci in the literature are insufficient, so it cannot be further stratified analysis. In spite of this, this study still ensures the truthfulness and reliability of the research results from the following points. First of all, this article included a large number of cases and controls from different studies, and the literature was collected until January 2021, involving Asian and White races, which significantly increased the statistical testing efficiency. What's more, for the same population source or repeatedly published literature, only the larger or recent data results are included in the study, thus ensuring that there is no obvious selection bias.

## Author contributions

**Conceptualization:** Ming Li, Zhongbao Hu.

**Data curation:** Zhongbao Hu, Jingsheng Chen.

**Formal analysis:** Ming Li, Ping Meng.

**Funding acquisition:** Ming Li.

**Investigation:** Zhongbao Hu.

**Methodology:** Ping Meng, Zhongbao Hu, Zhongbao Hu.

**Project administration:** Ming Li.

**Resources:** Jingsheng Chen.

**Software:** Jingsheng Chen.

**Supervision:** Jingsheng Chen, Ping Meng, Zhongbao Hu.

**Validation:** Zhongbao Hu, Jingsheng Chen, Ping Meng.

**Visualization & software:** Zhongbao Hu, Jingsheng Chen.

**Visualization:** Ping Meng.

**Writing – original draft:** Zhongbao Hu, Ming Li.

**Writing – review & editing:** Zhongbao Hu, Ming Li.
